# Modified Osteotome Sinus Floor Elevation Technique for Multiple Edentulous Spaces: A Non-Randomized Controlled Trial

**DOI:** 10.3390/ijerph19138019

**Published:** 2022-06-30

**Authors:** Ning Kang, Caojie Liu

**Affiliations:** 1 Department of Dental Implant, West China Hospital of Stomatology, Sichuan University, Chengdu 610041, China; 2 College of Biomedical Engineering, Sichuan University, Chengdu 610041, China

**Keywords:** sinus floor augmentation, dental implants, Schneiderian membrane, residual bone height, simultaneous implant placement

## Abstract

**Objectives:** We aimed to demonstrate our modified osteotome sinus floor elevation (OSFE) technique for placing two implants in multiple maxillary posterior edentulous spaces with residual bone height (RBH) < 5 mm, to evaluate the clinical effect and explore the prognosis. **Methods:** We identified 18 appropriate patients with RBH < 5 mm and 12 patients with RBH ≥ 5 mm. After drill preparation, variously shaped curettes were applied to adequately release the tension of the membrane around the cavity and between two implants by blunt dissection. Then, an osteotome was used to elevate the membrane to the desired height. After filling bone graft into the elevated space, dental implants were inserted. Cone-Beam Computed Tomography (CBCT) was performed after surgery and 6 months later. **Results:** The implant survival rate was 100%, and after the 6-month resorption, the height of the graft apically between the two implants gradually stabilized at 8.92 mm. Compared with 12 patients with RBH ≥ 5 mm, their graft bone resorption demonstrated no significant difference. **Conclusions:** It can be suggested that the modified OSFE technique could yield predictable clinical results for placing adjacent implants in patients with RBH less than 5 mm after six months of follow-up. **Clinical Significance:** Our modified OSFE technique could be applied to place adjacent implants in patients with RBH less than 5 mm, especially for elderly patients or patients with bone crests and vessels on the lateral wall, owing to its advantages including less trauma and fewer complications, minimizing the risk of membrane perforation, shortening the treatment period, avoiding another surgery area or second-stage surgery, improving not only the bone around the implant apex but also between implants, etc.

## 1. Introduction

Owing to periodontitis, decreased support and deficient occlusal force after tooth extraction, alveolar bone resorption is accelerated [1]. In addition, increased maxillary sinus pneumatization also contributes to the decrease in alveolar bone in the maxillary posterior area [2]. Inadequate bone height is a clinical challenge of implantation and prosthodontic treatment.

To create appropriate conditions for an implant, the operation of sinus floor elevation is an ideal strategy to restore sufficient alveolar bone height. In 1976, Tatum first described the operation of lateral sinus floor elevation (LSFE), which requires creating surgical access by preparing an opening window in the external maxillary sinus wall. Via the lateral passage, the Schneiderian membrane can be carefully peeled from the inner aspect of the sinus cavity. After filling the space between the elevated membrane and the maxillary sinus floor with osteogenic graft, implants can be placed after a delay or simultaneously. This access strategy has been a classic surgical method appropriate for most patients undergoing sinus floor elevation [3].

In 1994 Summers first demonstrated the procedure of osteotome sinus floor elevation (OSFE), a kind of transcrestal sinus floor elevation. After preparing the alveolar bone until the remaining height is less than 1–2 mm, by knocking the osteotome, the sinus floor and the Schneiderian membrane are pushed into the maxillary sinus. Bone graft would be simultaneously filled into the elevated space, and the implants would be inserted most often simultaneously [4]. Through continuous improvement, the indication of traditional OSFE has been gradually expanded. Therefore, traditional OSFE has been generally recognized as a preferred approach for patients with residual bone height (RBH) more than 5 mm [5,6].

According to the International Team for Implantology (ITI) treatment guide, RBH > 6 mm or ≤6 mm would be the criteria of operation method selection between LSFE and transcrestal approaches. In addition, RBH > 5 mm or ≤5 mm would be the criteria of selection between simultaneous or delayed implants placement [6,7,8]. Though it has broader indications, LSFE is also accompanied by more trauma and higher risk of complications, e.g., ecchymosis, infection or Schneiderian membrane perforation [9,10,11]. Therefore, modifying the traditional OSFE technique to broaden its indications and improving the treatment outcome would be necessary and meaningful.

Various studies have been conducted to improve the sinus floor elevation technique, e.g., comparing lateral and transcrestal sinus floor elevation techniques with RBH less than 4 mm or 5 mm, comparing different transcrestal sinus floor elevation techniques via osteotome or hydrodynamic ultrasonic device, evaluating the osteogenesis effect with or without bone graft materials. [12,13,14]. Few studies have evaluated the existence of Schneider membrane perforation after OSFE, which would complicate the OSFE surgery and affect the success rate of implants [15]. In particular, the alteration of the surface tension coefficient of the Schneiderian membrane while performing OSFE adjacent to edentulous spaces via different techniques might have an impact on the integrity of the Schneiderian membrane, which demands exploration and discussion.

According to a realistic situation, we decided to demonstrate our modified OSFE and to explore the prognosis of multiple maxillary posterior edentulous spaces with RBH < 5 mm. Our research on the modified OSFE aimed to present its three major innovations:

First, protecting the Schneiderian membrane by releasing tension.

Second, less trauma and less technique sensitivity than LSFE.

Third, reliable conclusions with statistical evidence.

Analysis and conclusion derived from this research can be taken as supporting an alternative clinical procedure or as reference in order to develop individualized treatment programs for specific patients.

## 2. Methods and Materials

We conducted this study in compliance with the appropriate EQUATOR guidelines, the CARE Guidelines: Consensus-based Clinical Case Reporting Guideline Development [16]. According to the <Quality Control of Clinical Trial of drugs> issued by China Food and Drug Administration in 2003, the <Ethical Review of Biomedical Research Involving Humans> promulgated by China’s Ministry of Health in 2007, the <Administrative measures for clinical application of medical technology> issued by China’s Ministry of Health in 2009, and the <Helsinki Declaration> revised in 2013, upon review by the Institutional Review Board of West China Hospital of Stomatology, Sichuan University, the ethical review opinions of this project are agreed. The ethical clearance approval number is WCHSIRB-D-2016-192.

### 2.1. Patients’ Characteristics

The patient population attending the West China Hospital of Stomatology, Sichuan University, between April 2016 and June 2019 was screened for eligibility. Patients that fulfilled the inclusion criteria were fully informed of the study aims and nature. Benefits, as well as any potential complications, were explained in detail. The patients that wanted to participate in the study read and signed the informed consent.

All preoperative, postoperative and subsequent visit details are gathered from medical history, operative note and Cone-Beam Computed Tomography (CBCT) data. RBH was measured as the height from the sinus floor to the alveolar crest on the coronal view of CBCT image.

According to the RBH, patients were divided into 3 groups: Group 0–4 mm, Group 4–5 mm, and Group 5–7 mm. Group 0–4 mm and Group 4–5 mm were operated upon via modified OSFE technique. According to the ITI treatment guide, Group 5–7 mm satisfied the indication of traditional OSEF and were operated upon via traditional OSFE.

### 2.2. Inclusion and Exclusion Criteria

With the purpose of gathering valid data, the following inclusion and exclusion criteria were formulated.

The inclusion criteria were as follows:

- Partially edentulous patients at the maxillary posterior region, including the second premolar and first and second molar sites requiring dental implants.
- Bone width at the bony crest level sufficient to allow the insertion of regular-diameter (4.1 mm) and wide-diameter (4.8 mm) dental implants maintaining at least 1.0 mm of buccal and lingual bone. This requires at least 6–7 mm bone width at the bony crest level.
- Those with RBH in dentition defect area less than 5 mm should be included into modified OSFE group.
- Those with RBH in dentition defect area more than 5 mm should be included into traditional OSFE group.

The exclusion criteria were as follows:

- Patients with serious diseases (e.g., cardiovascular and cerebrovascular diseases, liver or kidney diseases).
- Patients who underwent major operations in recent 3 years.
- Patients with blood system diseases (e.g., blood coagulation disorder, anemia, the increase or decrease in leukocyte count or thrombocytopenia).
- Patients with diabetes, osteoporosis, hypertension, hyperthyroidism, rheumatism, allergies to medications or food.
- Female patients in menstrual period or pregnancy.
- Heavy smokers (more than 20 cigarettes daily).
- Patients with symptoms of sinus disease (e.g., maxillary sinusitis, antracele).
- Poor compliance, without regular subsequent visit.

### 2.3. Surgical Procedure of Modified OSFE

The correspondence author Dr. Kang completed all the sinus floor elevation and the implant-inserting surgeries. Dr. Kang is the inventor of our modified OSFE technique, whose clinical experience and skilled surgical technique ensured the accuracy and consistency of our research.

After conventional intraoral and extraoral sterilization and draping, operation area was prepared with local-infiltration anesthesia by injecting Articaine Hydrochloride with adrenaline (PRIMACAINE ADRENALINE 1/100,000). After a crestal incision, mucoperiosteal flap was fully elevated. Meanwhile, blood was collected with syringe [17].

**Figure 1:**
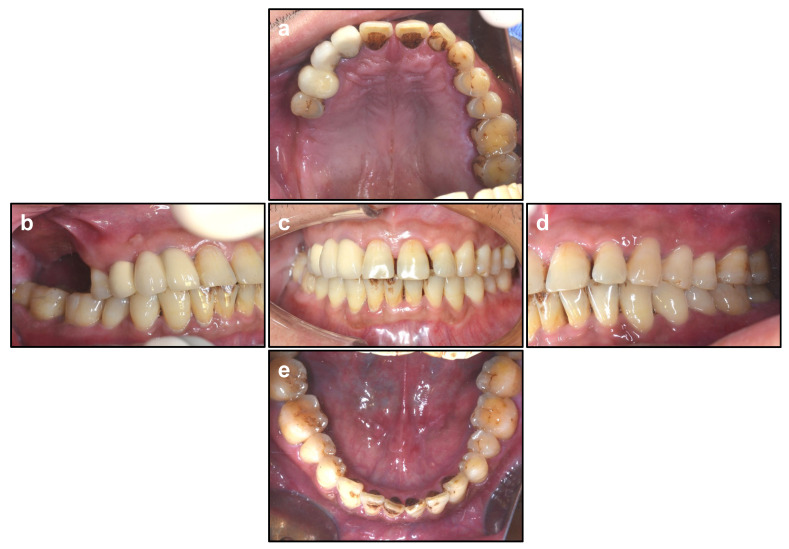
Occlusal Relationship. (**a**) Maxillary. (**b**) Right. (**c**) Occlusal. (**d**) Left. (**e**) Mandibular.

**Figure 2:**
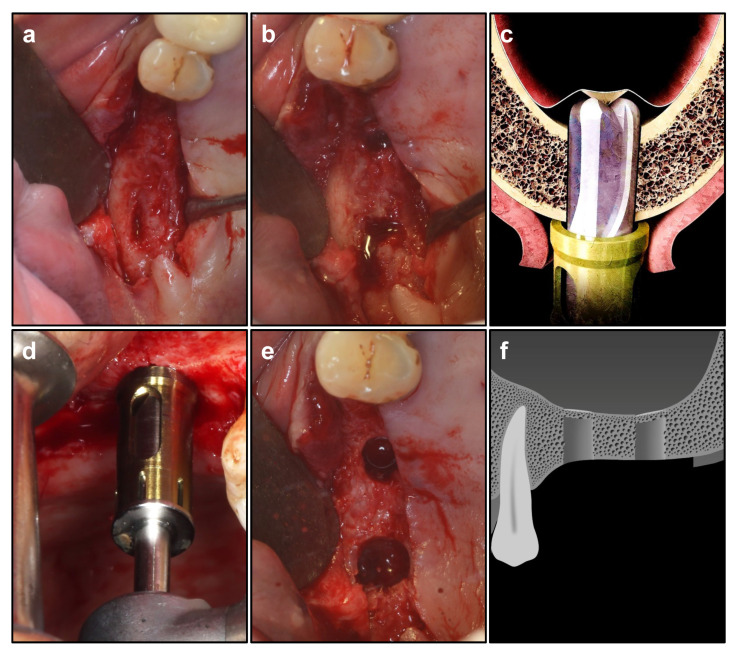
Sinus floor preparation. (**a**) performing H incision on teeth 16 and 17. (**b**) drill preparation. (**c**) Crestal Approach Sinus Kit (CAS) drill simulation. (**d**) using CAS drill for preparation. (**e**) integrated membrane after CAS drill preparation. (**f**) schematic diagram after drill preparation.

The implant location and orientation were determined according to occlusal relationship or surgical template (Figure 1). Pilot drill was used to drill 1 mm shorter than the remaining alveolar bone height [18]. Then, Crestal Approach Sinus Kit (CAS, Osstem Implant Co., Seoul, Korea) drills of different diameters and rounded tips were used in sequence to thoroughly grind the residual sinus floor bone until 1 mm bone was reserved in the sinus floor [19]. By applying the specialized stopper system, the risk of excessive drilling into the sinus cavity could also be avoided (Figure 2).

**Figure 3:**
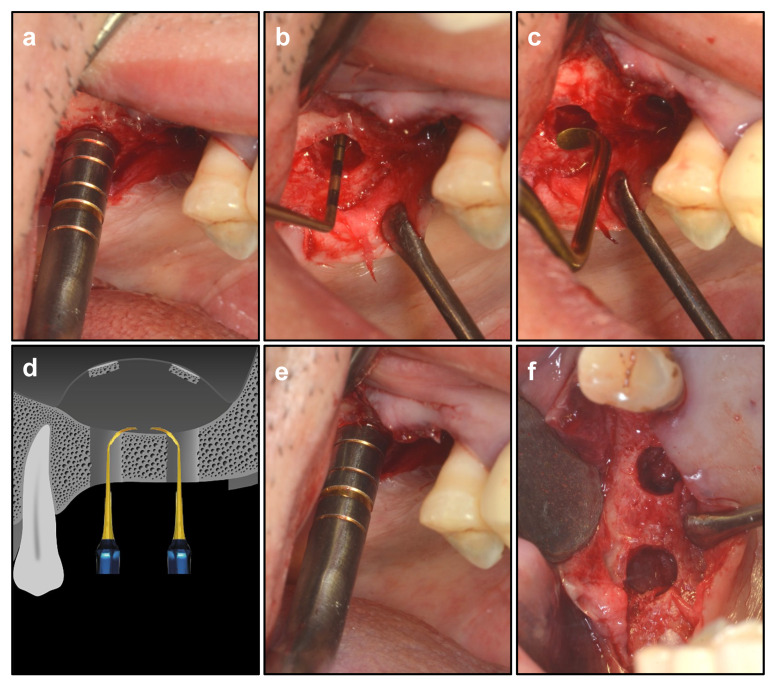
Schneiderian membrane elevation. (**a**) using osteotome to elevate the sinus membrane (>1 mm). (**b**) using dome-shaped sinus curette to lift the membrane. (**c**) using variously shaped detachers in Dentium Advanced Sinus Kit (DASK) to detach the membrane. (**d**) using detachers to elevate the membrane between 2 implants. (**e**) using osteotome to ascertain the height of elevation. (**f**) integrated membrane after elevation.

After preparation by CAS drills, the remaining 1 mm sinus floor was elevated by osteotomes (Bicon, Boston, MA, USA), and the elevated height was less than 1 mm. Such elevation height would create minor tension, which could ensure the integrity of the Schneiderian membrane [20]. Then, the dome-shaped sinus curette in Dentium Advanced Sinus Kit (DASK, Dentium, Korea) was used to gently release the tension of the sinus membrane around the cavity. Then, variously shaped detachers in DASK (REF XSE2/3/4 L) were applied to detach the Schneiderian membrane in all directions, in order to completely release the tension and create adequate space for osteogenic graft materials [21]. In particular, the membrane between 2 implants was carefully detached for bone formation in the middle space, with the aim of providing sufficient space for bone graft materials and strengthening the stability of 2 implants. Then, the osteotome was used to elevate the Schneiderian membrane without tension to the desired height and ascertain the height of elevation. The Valsalva maneuver was applied to check the integrality of the sinus membrane [22] (Figure 3).

**Figure 4:**
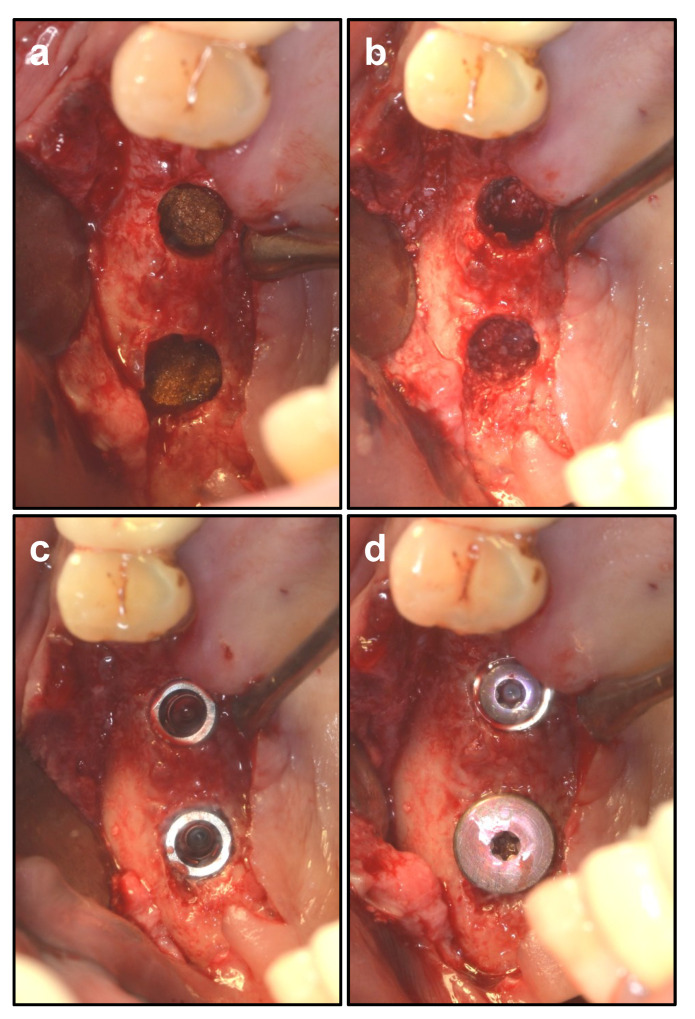
Guided bone regeneration (GBR) and inserting implants. (**a**) placing antibacterial Gelatamp into the sinus. (**b**) filling the space with Bio-Oss. (**c**) inserting the Implant. (**d**) placing a cover screw or a large-diameter healing cap.

Gelatamp (Coltene/Whaledent GmbH & Co. KG, Langenau, Germany) was inserted and carefully positioned to cover the sinus membrane on top of the implant and inter-implant sites. Small Geistlich Bio-Oss^®^ granules (particle size 0.25–1 mm) (Geistlich Pharma AG, Wolhusen, Switzerland) were mixed together with the blood collected before and then filled into the elevated space in sinus [23]. The area between 2 implants was also filled with Bio-Oss. Two implants were inserted into the prepared sinus floor [5,24]. The wound was sutured tightly (Figure 4).

**Figure 5:**
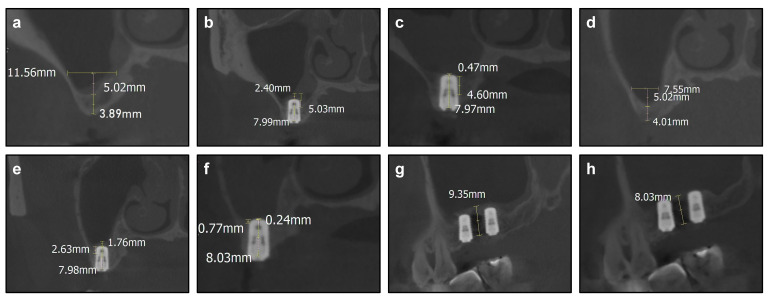
Cone-Beam Computed Tomography (CBCT) and measured data. (**a**) Residual bone height (RBH) of right maxillary first molar before elevation. (**b**) Height of the graft apically (aGH) of right maxillary first molar after elevation. (**c**) aGH of right maxillary first molar 6 months after elevation. (**d**) RBH of right maxillary second molar before elevation. (**e**) aGH of right maxillary second molar after elevation. (**f**) aGH of right maxillary second molar 6 months after elevation. (**g**) aGH between 2 implants after elevation. (**h**) aGH between 2 implants 6 months after elevation.

Patients immediately underwent CBCT to confirm the location of the implants and the effect of sinus membrane elevation. All patients received Amoxicillin and Clavulanate Potassium regularly for 3 days. Ten days later, the stitches were taken out, and 6 months later, patients underwent CBCT to determine the time for second-stage operation and prosthodontics (Figure 5).

### 2.4. Cone-Beam Computed Tomography Evaluations

The CBCT evaluation was designed according to Anchun Mo’s research [25]. CBCT was obtained at three time points during the study.

Time point T0: During evaluation of the future implant bed and implant planning.

Time point T1: Immediately after the sinus elevation and implant insertion.

Time point T2: Six months after the surgery before the second-stage surgery.

CBCT scanning was performed via 3 D Accuitomo 170 CBCT (J. MORITA CORP., Osaka, Japan) with the parameter at 80 kV, 4.5 mA. The scanning time was set as 23 s, and the voxel size was set as 0.125 mm in each dimension. The CBCT image reconstruction and analysis were performed in coronal and sagittal planes via the i-Dixel One Volume Viewer (Morita, Japan), with 1 mm slice interval.

**Figure 6:**
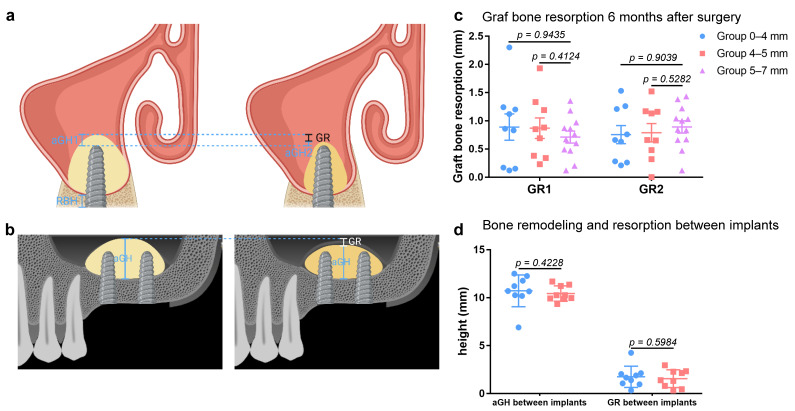
Analysis of bone remodeling and resorption. (**a**) schematic diagram of aGH and GR at implant site. (**b**) schematic diagram of aGH and GR between implants. (**c**) graft bone resorption at implant site 6 months after surgery. (**d**) bone remodeling and resorption between implants.

Measurement in the coronal plane was performed at the center of the implants at T1 and T2. Notably, measurement in the sagittal plane should be adjusted to cross the center of both the two implants at T1 and T2. Measurement at T0 should be adjusted to the same coronal or sagittal section of T1 and T2. The evaluated variables were measured as follows, with 0.01 mm measuring precision (Figure 6a,b).

RBH: the preoperative distance between the alveolar bone crest and the maxillary sinus floor in the coronal plane at T0.

Sinus width: the preoperative distance between the lateral sinus wall and the medial sinus wall at 5 mm height level from the sinus floor in the coronal plane at T0.

Height of the graft apically (aGH): the postoperative distance between implant apex and the elevated sinus floor in the coronal plane. aGH1 means the aGH at mesial implant site at T1, and aGH2 means the aGH at mesial implant site at T2. Correspondingly, aGH’1 means the aGH at distal implant site at T1, and aGH’2 means the aGH at distal implant site at T2.

aGH between implants: the postoperative distance between the cortical bone of maxillary sinus and the elevated sinus floor membrane in the sagittal plane.

Graft bone resorption (GR): the difference between aGH1 and aGH2, representing the resorbed graft bone. Correspondingly, GR between implants means the difference in aGH between implants at T1 and T2.

### 2.5. Statistical Analyses

All data were calculated as the mean ± standard deviation (SD) via Prism 7 (Version 7.04; GraphPad Software; 2365 Northside Dr., Suite 560, San Diego, CA, USA; https://www.graphpad.com/scientific-software/prism/, accessed on 16 September 2018). Before analyses, all data were tested for normality by Shapiro–Wilk normality test and homogeneity of variance. Statistical difference was analyzed via Student’s *t* test for independent sample test or one-way ANOVA with Tukey’s post hoc tests for multiple comparison. A *p* value < 0.05 was considered statistically significant.

## 3. Results

Between April 2016 and June 2019, we incorporated 30 patients into our research. Our case group was composed of 15 women and 15 men. The age of the participants ranged between 37 and 79 years. According to our grouping strategy, 9 patients were incorporated into Group 0–4 mm, 9 patients into Group 4–5 mm and 12 patients into Group 5–7 mm (Table 1).

No related complication or adverse effect was observed among those patients. Especially notably, no Schneiderian membrane perforation occurred during the operations. In general, the short-term success rate of these 30 patients was 100%. The RBH of patients ranged from 1.12 mm to 3.95 mm in Group 0–4 mm, from 4.01 mm to 4.97 mm in Group 4–5 mm and from 5.07 mm to 6.87 mm in Group 5–7 mm. The aGH of the 60 implant sites ranged from 0.66 mm to 4.96 mm at time point T1 and ranged from 0.24 mm to 3.84 mm at T2. The GR ranged from 0 to 2.3 mm (Table 2). According to the ANOVA and post hoc tests, there is no statistical difference (*p* > 0.05) in the GR of the three groups (Figure 6c).

The aGH between implants ranged from 5.07 mm to 12.5 mm at T1 and ranged from 5.02 mm to 10.83 mm. The GR between implants ranged from 0.33 mm to 4.26 mm (Table 3). Because the site between implants was not elevated in Group 5–7 mm, aGH and GR between implants in Group 5–7 mm were not included into the comparison. According to Student’s *t* test, GR between implants demonstrates no statistical difference (*p* > 0.05) between Group 0–4 mm and Group 4–5 mm (Figure 6d).

## 4. Discussion

A series of existing studies with respect to sinus floor elevation have declared different techniques for patients with different RBH [20]. These studies have provided insight and concepts for researching methods and statistical analysis. However, we noticed that there are hardly any articles researching the effect of inserting two implants in multiple maxillary posterior edentulous spaces with RBH < 5 mm [19]. In actuality, this is also a common phenomenon and a frequent requirement in clinical work. The great majority of previous researchers also emphasized that their studies only used short implants. Therefore, their conclusions might not be suitable for patients choosing normal-sized implants [26]. Our surgical procedures are in accordance with the guidelines, beyond partial adjustments to the procedure of membrane elevation, to make the procedures safer and more convenient [27].

### 4.1. Analysis of the Outcomes via Modified OSFE

As demonstrated in Table 2, after the 6-month resorption, the aGH between implants in Group 0–4 mm and Group 4–5 mm gradually stabilized at 8.92 mm. The elevated height between implants approximately fit the average length of the inserted implants, 9.16 mm, which concurs with the theory that the elevated height after resorption is determined by the inserted implant length [28]. Such space was maintained as a tent by the two implants. After gently detaching the Schneiderian membrane and filling bone graft, said tent tends towards dimensional stability and gradual ossification, which would provide mechanical support for the two implants.

The tendencies of aGH and GR correspond to the stable osteogenic effect. According to the analysis of the data, we can basically observe the predictable clinical results for applying modified OSFE technique to continuously place two implants simultaneously with RBH < 5 mm. Namely, the graft bone resorption in 6 months showed comparable levels in patients with different RBH. Further, patients with poor RBH, even RBH < 4 mm, could maintain similar graft bone resorption level to patients with RBH ≥ 5 mm via modified OSFE.

### 4.2. Technique Essentials of Modified OSFE

We aimed to achieve minimally invasive surgery and satisfactory outcomes with modified OSFE. This procedure has the advantages of less trauma and hemorrhage, less pain and more rapid recovery than LSFE, but accordingly, there exist some technical difficulties. How can tension of the Schneiderian membrane be relieved, in order to obtain more elevating space? How can the integrity of the membrane be ensured? How can adequate primary stability be obtained with limited RBH?

As reported in a previous study, the sinus membrane perforation rate would be 28% when RBH is less than 5 mm [29]. Notably, the perforation always occurred during the operation, and the perforation position was mostly at the central of elevated membrane, where the membrane became relatively thin.

According to previous research, the stress of the Schneiderian membrane can be calculated in a series of three-dimensional finite-element models [30]. By applying elongation test, perforation test, etc., researchers simulated and analyzed the dynamic parameters during Schneiderian membrane detachment. The conclusion was drawn that the maximum deformation can be mainly observed at the center of the separating membrane, and the maximum stress was detected at the margin of the detached membrane.

The following parameters were defined: F = axial load, A = cross-section area, A_s_ = shear-stress area, R = inside radius, T = thickness of the membrane, *σ =* axial stress, τ = shear stress.

(1) $$\sigma =\frac{F}{A}=\frac{F}{\pi R^{2}}$$

(2) $$\tau =\frac{F}{A_{s}}=\frac{F}{T2\pi R}$$

According to the function, it could be inferred by deduction that both axial stress and shear stress would decrease with the increase in the inside radius. Concerning this issue, we applied the dome-shaped sinus curette in DASK to gently lift the sinus membrane and variously shaped detachers in DASK to detach the Schneiderian membrane in all directions.

Apparently, by applying the modified OSFE technique, the Schneiderian membrane covered on adjacent implant sites can be detached into a lager circle or ellipse with lager radius than a single implant site. Consequently, the modified OSFE technique can release the tension of the Schneiderian membrane, both axial stress and shear stress. Therefore, the risk of perforation could be effectively reduced. As shown in the operation result, the implant survival rate was 100% without one case of Schneiderian membrane perforation.

Gelatamp would expand after absorbing blood, supporting and protecting the elevated membrane, analogous to a barrier membrane. Additionally, Gelatamp could slowly release silver ion (Ag^+^) to enhance its antibacterial function [31].

As for the primary stability, we applied the special design of CAS drills to reserve segmental sinus floor, because such autogenous bone is beneficial to implant stability. The specially designed round shape of the CAS drill top enhances the convenience of operation. It could ensure the safety of the Schneiderian membrane while drilling, forming conical bone slices and producing bone granules [4,32]. The elevated bone fragment can support and maintain the space, and double cortical bone can contribute to retention and stabilization of the implant. Additionally, the osteoinduction of autogenous bone could also facilitate osteogenesis.

### 4.3. Innovations and Advantages in Modified OSFE

As reviewed in the introduction, RBH ≤ 6 mm was suggested as a criterion to choose LSFE by the ITI treatment guide [6]. Consequently, simultaneously placing two implants in multiple maxillary posterior edentulous spaces with RBH < 5 mm via lateral or transcrestal approach is quite a challenge for the surgeon.

In fact, however, such RBH is quite common in elderly patients, especially patients with dentition defects owing to periodontitis. Furthermore, the anatomical structure in maxillary sinus could be complicated. Bone crests and vessels on the lateral wall or on the floor can be intractable obstacles for LSFE [33]. While applying LSFE to patients with wide maxillary sinus, it would be difficult to dissect the Schneiderian membrane with corresponding instruments [34]. When filling bone graft through the lateral window, it would form a slope in the sinus, which would be inappropriate for angiogenesis and implant osseointegration [35]. Additionally, the disadvantages of LSFE are also inevitable, e.g., higher risk of sinus membrane perforation [36]. The lateral operating area is also an additional wound, which may cause a series of complications, longer recovering period and higher risk of infection [37]. According to a prospective study by Carlo Rengo, 34.21% to 44.74% of patients reported severe pain during the first 2 days after LSFE surgery. Within a week after surgery, 97.36% of the patients experienced swelling, and 51.32% of the patients experienced ecchymosis [9]. In consideration of the trauma and possible complications, such wounds would be tremendous burdens for elderly patients.

Therefore, our clinical group was devoted to modifying the OSFE, in order to ensure the implant stability and reduce trauma and complications. Compared with LSFE, our modified OSFE technique based on CAS Kit and DASK is evaluated to be less invasive, because it avoids extra operating area and additional trauma [38]. Patients will undergo less operating time and less postoperative recovery time, and the probability of complications has also decreased accordingly [38].

What, then, are the advantages and innovations of our modified OSFE technique compared with tradition OSFE? Traditional OSFE has recently been the most widely used procedure in hospital, but it still has several deficiencies [14]. Knocking the osteotome might arouse a series of maxillary sinus complications [39]. The risk of puncturing the sinus membrane mostly occurs in the knocking procedure, especially for the patients with poor RBH [40]. Previous research from Tetsuya Sonoda has demonstrated their two-stage approach for patients with RBH between 4–6 mm, which has been proved effective and with relatively low risk of Schneiderian membrane perforation [17]. However, the limitations of this approach are also apparent, including the prolonged treatment period, the additional trauma caused by the second surgery and the potential for thinning the Schneiderian membrane.

One of the most important innovations in our modified OSFE technique is the adequate release of the Schneiderian membrane by using detachers to carefully dissect the sinus membrane. This step could completely avoid the risk of membrane perforation and would not lead to the thinning Schneiderian membrane, which explained why there occurred no membrane perforation among our 30 patients, including 9 patients with RBH between 4–5 mm and even 9 patients with RBH < 4 mm. The membrane between the two implants was carefully elevated to provide more sufficient area for osteogenesis between the two implants. Traditional OSFE always formed two individual tents in the sinus, and the area between two implants was separated by membrane [3]. This technique would peel the Schneiderian membrane entirely over two implants with detachers. Bone graft could also ossify between the two implants, which not only fulfilled the standard 3 mm distance between the two implants but also provided more bone support for implants. Thus, this technique contributed to the post-operative stability for the two implants.

In sum, owing to the above-mentioned innovations in our OSFE technique, its advantages are apparent, including but not limited to less trauma and fewer complications, minimizing the risk of membrane perforation, shortening the treatment period, avoiding another surgery area or second-stage surgery and improving not only the bone around the implant apex but also between implants. Therefore, our modified OSFE technique could be applied to place adjacent implants in patients with RBH < 5 mm, especially for patients who could hardly withstand more invasive operations, such as LSFE.

### 4.4. Limitations

There are several limitations in our study. First, to evaluate the effect of the implantation, five-year survival rate and ten-year survival rate are significant indicators, so long-term evaluation is still being carried out and will be reported in our follow-up studies. Furthermore, an point at which to innovate upon this research is to elevate and fill the space between two implants. We attempted to connect the osteogenesis between the two implants and implant stability. However, lacking in animal models, we do not have intuitive evidence to visualize the relationship between the two variables. Thus, this research will continue, both in long-term follow-up and in vivo animal models, in order to trace its influence on the osteogenesis implant stability.

## 5. Conclusions

Based on the results of this study, it can be suggested that the modified OSFE technique could yield predictable clinical results for continuously placing two implants simultaneously with RBH < 5 mm.

Our study incorporated 18 cases with modified OSFE technique, and the survival rate was 100%. After 6-month recovery time, all cases were evaluated to be successful from CBCT measurement. Further analysis indicated no significant difference was observed among the graft bone resorption in the three groups. Thus, the indication of the modified OSFE can be expanded to patients with RBH from 0–5 mm.

According to clinical application, the modified OSFE procedure is more applicable to patients with RBH < 5 mm and has the advantages of less trauma and hemorrhage, less pain and more rapid recovery. Therefore, this technique may reduce the complications of complex invasive procedures and simplify the treatment in multiple maxillary posterior edentulous spaces.

Within the limitations of this study, it can be suggested that the modified OSFE technique could yield predictable clinical results for placing adjacent implants in patients with RBH less than 5 mm after six months of follow-up.

## Figures and Tables

**Table 1:** Preoperative patient and bone characteristics.

Group 0–4 mm	Sex	Age (Year)	Implant Site	RBH (mm)	Sinus Width (mm)	Implant Site’	RBH’ (mm)	Sinus Width’ (mm)
No.1	Male	45	15	3.49	17.31	16	2.56	9.96
No.2	Female	65	15	1.12	14.99	16	2.12	14.99
No.3	Female	55	26	3.69	14.42	27	2.64	11.41
No.4	Male	42	26	3.72	13.75	27	3.95	11.98
No.5	Male	42	15	2.50	16.89	16	2.14	14.51
No.6	Male	41	26	3.25	12.33	27	2.07	9.38
No.7	Male	53	16	2.60	11.15	17	1.71	16.52
No.8	Female	49	26	3.54	10.62	27	3.89	8.91
No.9	Male	54	26	1.66	14.33	27	3.27	12.96
Average ± SD		49.6 ± 7.97	-	2.84 ± 0.94	13.98 ± 2.32	-	2.71 ± 0.82	12.29 ± 2.66
**Group 4–5 mm**								
No.10	Female	49	16	4.65	11.56	17	4.01	7.55
No.11	Male	41	16	4.78	12.37	17	4.82	11.68
No.12	Male	47	16	4.51	11.86	17	4.17	12.95
No.13	Male	77	16	4.08	15.13	17	4.64	14.05
No.14	Female	79	26	4.95	12.17	27	4.16	13.5
No.15	Female	68	15	4.87	12.78	16	4.49	9.62
No.16	Female	37	15	4.89	15.51	16	4.97	10.42
No.17	Female	55	15	4.93	12.91	16	4.11	10.13
No.18	Female	57	15	4.27	15.21	16	4.94	9.13
Average ± SD		56.7 ± 15.11	-	4.66 ± 0.31	13.28 ± 1.56	-	4.48 ± 0.38	12.29 ± 2.66
**Group 5–7 mm**								
No.19	Male	63	25	5.24	12.62	26	5.09	13.15
No.20	Female	54	26	6.73	13.86	27	6.4	11.77
No.21	Female	56	15	5.39	13.02	16	6.61	11.98
No.22	Female	62	16	6.7	13.95	17	6.22	15.36
No.23	Male	72	16	6.55	13.63	17	6.42	11.27
No.24	Male	41	26	6.72	11.64	27	6.7	11.52
No.25	Male	40	26	5.42	15.49	27	5.37	15.31
No.26	Female	48	16	5.97	14.69	17	5.07	13.81
No.27	Female	53	26	5.37	15.61	27	5.13	15.95
No.28	Male	57	15	6.87	14.16	16	6.99	15.62
No.29	Female	63	26	5.44	14.17	27	5.58	11.5
No.30	Male	52	25	6.68	11.89	26	6.86	13.87
Average ± SD		55.1 ± 9.32	-	6.09 ± 0.67	13.73 ± 1.26	-	6.04 ± 0.74	13.43 ± 1.8

RBH (residual bone height); SD (standard deviation). The indicators without ’ represent the mesial implant site, e.g., implant site, RBH, sinus width. The indicators with ’ represent the distal implant site, e.g., implant site’, RBH’, sinus width’. The implant sites are recorded by Fédération Dentaire Internationale (FDI) dental notation.

**Table 2:** Surgery documents and CBCT measurement of implant site.

Group 0–4 mm	Time of Surgery	Implant Size (mm)	Primary Stability (Ncm)	aGH1 (mm)	aGH2 (mm)	GR (mm)	Implant Size’ (mm)	Primary Stability’ (Ncm)	aGH1′ (mm)	aGH2′ (mm)	GR’ (mm)
No.1	26 July 2017	4.8 × 8	20	2.43	1.23	1.2	4.8 × 8	5	1.97	0.44	1.53
No.2	14 December 2017	4.1 × 10	30	2.07	0.83	1.24	4.8 × 8	20	1.83	1.57	0.26
No.3	17 February 2018	4.8 × 10	5	1.97	1.14	0.83	4.8 × 10	5	0.66	0.45	0.21
No.4	25 January 2018	4.8 × 10	20	1.60	0.74	0.86	4.8 × 8	35	3.97	3.21	0.76
No.5	29 January 2018	4.1 × 10	30	2.42	2.3	0.12	4.8 × 8	10	1.12	0.49	0.63
No.6	11 July 2017	4.8 × 10	20	1.29	1.12	0.17	4.8 × 8	20	4.08	2.82	1.26
No.7	13 April 2016	4.8 × 8	20	4.96	3.84	1.12	4.8 × 8	20	4.24	3.58	0.66
No.8	3 November 2017	4.8 × 8	35	2.65	2.5	0.15	4.8 × 8	35	1.74	0.53	1.21
No.9	31 August 2017	4.8 × 8	30	2.96	0.66	2.3	4.8 × 8	30	1.92	1.64	0.28
Average ± SD	-	-	23.33 ± 9.01	2.48 ± 1.06	1.6 ± 1.07	0.89 ± 0.7	-	20 ± 11.73	2.39 ± 1.35	1.64 ± 1.28	0.76 ± 0.48
**Group 4–5 mm**											
No.10	15 November 2017	4.8 × 8	35	2.4	0.47	1.93	4.8 × 8	35	1.76	0.24	1.52
No.11	11 July 2018	4.8 × 10	20	1.87	1.53	0.34	4.8 × 8	10	2.13	1.47	0.66
No.12	1 April 2019	4.8 × 10	30	1.68	0.99	0.69	4.8 × 10	25	2.92	2.27	0.65
No.13	12 April 2019	4.8 × 10	25	2.36	1.51	0.85	4.8 × 10	35	1.53	1.05	0.48
No.14	12 April 2019	4.8 × 10	20	2.75	1.42	1.33	4.8 × 10	30	2.56	2.24	0.32
No.15	19 April 2019	4.1 × 10	20	1.65	1.42	0.23	4.8 × 10	25	2.44	1.31	1.13
No.16	23 April 2019	4.1 × 8	15	1.9	1.02	0.88	4.8 × 10	35	2.18	1.01	1.17
No.17	20 June 2019	4.1 × 10	20	1.76	1.38	0.38	4.8 × 10	5	1.13	1.13	0
No.18	21 June 2019	4.1 × 10	25	2.9	1.71	1.19	4.8 × 10	25	2.75	1.59	1.16
Average ± SD	-	-	23.33 ± 6.12	2.14 ± 0.47	1.27 ± 0.38	0.87 ± 0.55	-	25 ± 10.9	2.16 ± 0.9	1.37 ± 0.63	0.79 ± 0.49
**Group 5–7 mm**											
No.19	27 December 2018	4.1 × 10	35	1.77	1.35	0.12	4.8 × 10	30	2.17	1.17	1
No.20	11 January 2019	4.8 × 10	30	2.13	0.86	0.97	4.8 × 10	35	1.97	1.31	0.66
No.21	31 January2019	4.1 × 8	10	2.08	1.02	0.76	4.8 × 8	10	2.88	1.66	1.22
No.22	2 February 2019	4.8 × 10	15	1.69	0.9	0.49	4.8 × 10	30	2.07	1.23	0.84
No.23	12 February 2019	4.8 × 10	30	2.67	1.77	0.6	4.8 × 10	30	2.7	1.32	1.38
No.24	14 February2019	4.8 × 10	15	2.06	1.29	0.47	4.8 × 10	30	1.87	1.2	0.67
No.25	14 February2019	4.1 × 8	35	2.55	1.07	1.18	4.1 × 8	15	2.83	1.79	1.04
No.26	21 February2019	4.1 × 8	20	2.64	1.48	0.86	4.8 × 8	25	1.82	1.35	0.47
No.27	5 March 2019	4.1 × 10	15	2.54	1.37	0.87	4.8 × 10	30	2.74	1.31	1.43
No.28	18 March 2019	4.8 × 8	30	2.53	0.88	1.35	4.8 × 10	30	2.26	1.23	1.03
No.29	26 March 2019	4.8 × 10	10	2.11	1.61	0.2	4.8 × 8	25	2.21	1.38	0.83
No.30	7 April 2019	4.8 × 8	25	2.61	1.64	0.67	4.8 × 10	35	1.7	1.58	0.12
Average ± SD	-	-	22.5 ± 9.41	2.28 ± 0.35	1.27 ± 0.32	0.71 ± 0.37	-	27.08 ± 7.53	2.27 ± 0.42	1.38 ± 0.2	0.89 ± 0.38

aGH (height of the graft apically); GR (graft bone resorption); SD (standard deviation). The indicators without ’ represent the mesial implant site, e.g., implant size, primary stability, aGH1, aGH2, GR. The indicators with ’ represent the distal implant site, e.g., implant size’, primary stability’, aGH1′, aGH2′, GR’. The implants’ sizes are recorded as diameter × length (mm).

**Table 3:** CBCT Measurement of the site between implants.

Group 0–4 mm	aGH between Implants (mm)	aGH between Implants (mm)	GR between Implants (mm)
No.1	10.25	8.12	2.13
No.2	11.21	10.14	1.07
No.3	12.50	10.83	1.67
No.4	11.77	9.72	2.05
No.5	10.18	5.92	4.26
No.6	12.26	10.37	1.89
No.7	10.65	9.26	1.39
No.8	10.69	9.72	0.97
No.9	6.91	6.58	0.33
Average ± SD	10.71 ± 1.66	8.96 ± 1.72	1.75 ± 1.11
**Group 4–5 mm**			
No.10	9.35	8.03	1.32
No.11	10.26	8.87	1.39
No.12	11.22	8.94	2.28
No.13	9.92	9.12	0.8
No.14	9.86	7.71	2.15
No.15	10.26	9.93	0.33
No.16	11.68	9.35	2.33
No.17	9.96	9.51	0.45
No.18	11.36	8.41	2.95
Average ± SD	10.43 ± 0.8	8.87 ± 0.72	1.56 ± 0.92

aGH (height of the graft apically); GR (graft bone resorption); SD (standard deviation).

## Data Availability

The datasets used and analyzed during the current study are available from the corresponding author on reasonable request.
